# Rezafungin exhibits anti-biofilm properties against fungal biofilms *in vitro*

**DOI:** 10.1093/jac/dkag058

**Published:** 2026-03-05

**Authors:** Hafsa Abduljalil, Kerry Bartie, Abhijit M Bal, Riina Rautemaa-Richardson, Craig Williams, Ryan Kean, Gordon Ramage

**Affiliations:** Safeguarding Health Through Infection Prevention (SHIP) Research Group, Research Centre for Health, School of Health and Life Sciences, Glasgow Caledonian University, 70 Cowcaddens Road, Glasgow G4 0BA, UK; Safeguarding Health Through Infection Prevention (SHIP) Research Group, Research Centre for Health, School of Health and Life Sciences, Glasgow Caledonian University, 70 Cowcaddens Road, Glasgow G4 0BA, UK; Microbiology Department, Glasgow Royal Infirmary, Glasgow G4 0SF, UK; Mycology Reference Centre Manchester, ECMM Centre of Excellence, and Department of Infectious Diseases, Wythenshawe Hospital, Manchester University NHS Foundation Trust, UK; and Manchester Biomedical Research Centre, Division of Evolution, Infection and Genomics, Faculty of Biology, Medicine and Health, University of Manchester, Manchester, UK; Microbiology Department, Lancaster Royal Infirmary, Lancaster, UK; Safeguarding Health Through Infection Prevention (SHIP) Research Group, Research Centre for Health, School of Health and Life Sciences, Glasgow Caledonian University, 70 Cowcaddens Road, Glasgow G4 0BA, UK; Safeguarding Health Through Infection Prevention (SHIP) Research Group, Research Centre for Health, School of Health and Life Sciences, Glasgow Caledonian University, 70 Cowcaddens Road, Glasgow G4 0BA, UK

## Abstract

**Objectives:**

We sought to evaluate the comparative activity of rezafungin compared with caspofungin and other antifungal classes against biofilms from a large clinical panel of *Candida* strains (*n* = 167).

**Methods:**

Biofilm killing and inhibition were assessed using standard XTT [2,3-bis(2-methoxy-4-nitro-5-sulfophenyl)-2H-tetrazolium-5-carboxanilide salt] metabolic assessment. Biofilm time–kill kinetics were also evaluated using metabolic and viable cell counts. Microscopy was performed to visually assess biofilm inhibition.

**Results:**

Rezafungin was shown to outperform caspofungin and other antifungals against *C. albicans*, *C. parapsilosis*, *C. tropicalis* and *Nakaseomyces glabratus* (previously called *C. glabrata*) strains with a heterogeneous biofilm phenotype. Assessment of high biofilm-forming strains at 0.03 mg/L concentrations showed that rezafungin killed biofilms to an equal or greater extent than caspofungin. Time–kill studies showed a rapid reduction in metabolism and viable cfus by both rezafungin and caspofungin, but with little difference between both compounds. Evaluation of biofilm inhibition characteristics of both compounds showed that rezafungin was marginally more effective than caspofungin, which was corroborated by microscopical analyses.

**Conclusions:**

Together, these data show that rezafungin is non-inferior to caspofungin in terms of anti-biofilm activity and displays characteristics that suggest it can control biofilms more effectively than caspofungin. Further evaluation is required to establish whether these *in vitro* effects translate clinically, but the data indicate an opportunity for rezafungin to be used for the clinical management of biofilm-related diseases.

## Introduction

Biofilms are matrix-enclosed populations of microbes, bacteria and fungi that coalesce as large surface-attached structures or aggregates, which may or may not be associated with host tissue.^1^ Fungal biofilms are important clinical entities typified by their resilient phenotype during treatment with antifungal agents.^2^  *Candida* species, and in particular *C. albicans*, have been the most studied in the context of biofilm development and associated attributes. These are critically important in bloodstream infections (BSIs), where the biofilm phenotype has been shown to correlate with significantly worse clinical outcomes.^3,4^ Our group previously reported that *C. albicans* was isolated from 41% of patients from a Scottish BSI cohort, which was followed closely by *Nakaseomyces glabratus* (35%)—data that are consistent with the epidemiology in Europe.^5^ In our study we were able to demonstrate a positive correlation between mortality and high levels of *C. albicans* biofilm formation.^6,7^ Indeed, differential biofilm-forming abilities and more general strain heterogeneity appear to be important drivers of clinical outcomes.^8^

Clinical guidelines indicate the use of echinocandins as first-line therapy for the management of line-related BSI, alongside catheter removal.^9^ This class of compounds target the β-1,3-D-glucan synthase pathway by inhibiting Fks1p.^10^ The earliest evidence for the clinical utility of managing *C. albicans* biofilms came from studies of caspofungin, where potent anti-biofilm activity was demonstrated.^11–13^ As newer echinocandins, such as micafungin and anidulafungin, were developed, modest *in vitro* class differences were observed,^14^ but without any significantly improved anti-biofilm-related effects.^15,16^ Studies have shown that when no genetic differences in *FKS1* mutations are observed then it is possible that subtle structural differences among echinocandins may affect binding, which accounts for variable efficacy across species.^14^ Despite echinocandins being large complex molecules that are not overly lipophilic, and although there remain questions about tolerance and heteroresistance associated with echinocandins, they remain a gold standard in managing candidaemia.^17^

Rezafungin is the newest echinocandin licensed for clinical use. Due to its very long half-life of approximately 130 h, compared with 9–11 h for caspofungin, it can be used as a once-weekly infusion. In clinical trials, it has been shown to be non-inferior compared with caspofungin for all-cause mortality.^18,19^ Despite these promising data, there is limited evidence for anti-biofilm activity. *In vitro* studies report a significant prevention of biofilm development and eradication of preformed mature biofilms, albeit against a limited number of strains.^20,21^ There is also evidence of activity in invasive disease in animal studies.^22^

With the advent of rezafungin, we hypothesized that this new echinocandin could be equivalent to caspofungin. This study aimed to undertake a large-scale approach to assess rezafungin anti-biofilm activity compared with caspofungin. Here, we demonstrate the non-inferiority of rezafungin and evidence of its ability to effectively inhibit biofilm formation.

## Materials and methods

### Strain propagation, maintenance and antifungals

A panel of *Candida* species from a Scottish candidaemia study were used, consisting of *C. albicans* (*n* = 113), *N. glabratus* (*n* = 34), *Candida parapsilosis* (*n* = 10), *Candida tropicalis* (*n* = 10), along with *C. albicans* SC5314,^6,7^ and containing isolates that have been extensively studied elsewhere.^23,24^ All strains were prepared on Sabouraud dextrose (Sigma-Aldrich, Dorset, UK) for 48 h in aerobic conditions and refrigerated at 4°C prior to proliferation in yeast peptone dextrose (Sigma-Aldrich, Dorset, UK) in a 200 rpm shaking incubator, overnight, at 30°C. Yeast cells were pelleted through centrifugation at 3000 rpm for 5 min and washed twice with PBS (Sigma-Aldrich, Dorset, UK). Cells were then counted using a Neubauer haemocytometer and standardized to a working concentration for further experimentation. A series of 2-fold dilutions of rezafungin (Napp Pharmaceuticals), caspofungin (Sigma, Poole, UK), amphotericin B (Sigma, Poole, UK) and fluconazole (Sigma, Poole, UK) were prepared in 96-well round- or flat-bottomed microtitre plates (Corning Inc., NY, USA) at varying concentrations.

### Planktonic and sessile MIC testing

Planktonic MICs (PMICs) were determined using a broth microdilution method according to the M27-A3 standard for fungi with rezafungin, caspofungin, amphotericin B and fluconazole, ranging from 0.03 to 16 mg/L.^25^ Briefly, cells were adjusted to the desired density of 2 × 10^4 ^cells/mL in Roswell Park Memorial Institute (RPMI) medium (Sigma-Aldrich, Dorset, UK). The plates were incubated at 37°C. After 24 h, the MIC was determined as the lowest concentration that prevented visible growth.

For sessile MICs (SMICs) all isolates were standardized to the desired cellular density of 1 × 10^6 ^cells/mL into RPMI-1640 medium and the biofilms formed onto pre-sterilized, polystyrene, 96-well flat-bottomed microtitre plates as previously described.^26,27^ The plates were incubated at 37°C for 24 h (mature biofilms). After incubation, the biofilms were washed with PBS to remove the loosely attached cells, and serial doubling dilutions of each antifungal (rezafungin, caspofungin, amphotericin B and fluconazole) prepared ranging from 0.03 mg/L to 16 mg/L. Endpoint SMICs were assessed after incubation for 24 h at 37°C. Treated biofilms were washed with PBS, and the XTT [2,3-bis(2-methoxy-4-nitro-5-sulfophenyl)-2H-tetrazolium-5-carboxanilide salt] (Sigma-Aldrich, UK) metabolic reduction assay performed immediately after washing, to assess cell viability, as described previously.^27^ SMICs were calculated as the concentration where a 50% and 80% reduction (SMIC_50_ and SMIC_80_) in metabolism was calculated compared with the positive control. Positive control data from each isolate were used to assess the spread of biofilm formation for each isolate, and to select specific high and low biofilm-forming strains in subsequent assays. Assays were performed in triplicate.

Biofilm time–kill studies were also performed to compare rezafungin and caspofungin over 2, 4, 6 and 24 h for a select number of strains [five high biofilm formers (HBFs) and five low biofilm formers (LBFs)] were prepared as mature 24 h biofilms. Rezafungin and caspofungin were added at 0.125 and 0.25 mg/L (equivalent to 1× and 2× the PMIC_90_). The metabolic activity of each biofilm at each timepoint was then assessed using the XTT assay, as described above. We determined colony forming units (cfu) according to the Miles and Misra method. Briefly, biofilms of four *C. albicans* isolates (two HBFs and two LBFs) were grown on Thermanox^™^ coverslips for 24 h. Rezafungin and caspofungin (0.125 and 0.25 mg/L) were added to the mature biofilms and cfus were determined at 4 and 24 h. Coverslips were sonicated for 10 min at 35 Hz in 1 mL of PBS to disrupt the biofilms. The resulting sonicate was serially diluted (10-fold dilutions), and aliquots of each dilution were plated onto Sabouraud agar and incubated at 30°C for 48 h prior to cfu enumeration.

### Biofilm inhibition studies

For biofilm inhibition, a selection of the highest and lowest biofilm-forming *C. albicans* strains were standardized to 1 × 10^6^ cells/mL in RPMI-1640 according to established methods.^27^ Cells were then dispensed into 96-well flat-bottomed microtitre plates, followed by the addition of a final concentration of 0.007 to 4 mg/L of rezafungin and caspofungin. Cells were incubated for 24 h at 37°C and washed with PBS to remove non-adherent cells. The biofilm inhibitory effect was assessed and quantified using an XTT assay, as described above, quantifying an 80% reduction in biofilm viability based on XTT metabolic activity. Light microscopy was also performed to visualize the impact on biofilm inhibition using an EVOS microscope.

### Statistical analysis and data presentation

Graphs were produced using GraphPad Prism (Version 8.4.3; GraphPad Software Inc., La Jolla, CA). Data were tested for normal distribution using the D'Agostino–Pearson omnibus normality test. For statistical analysis, two-tailed Student’s *t*-test was used to compare the means of untreated controls and treated biofilms. One-way analysis of variance (ANOVA) with Tukey’s post-test was used to compare data of more than two samples for 24 h biofilm inhibition. For this percentage metabolic activity data were converted to proportions and log_10_ transformed prior to analysis, and a two-way ANOVA performed for analysis of time–kill.

## Results

### Rezafungin is effective against planktonic *Candida*

Rezafungin showed the greatest *in vitro* activity against *C. albicans* (*n* = 113), with MIC_50_ ≤ 0.03 mg/L and MIC_90_ = 0.06 mg/L (range ≤0.03 to 0.5 mg/L). Caspofungin demonstrated slightly higher MICs (MIC_50_ = 0.06 mg/L; MIC_90_ = 0.125 mg/L), followed by fluconazole and amphotericin B, which showed progressively reduced activity. Planktonic MIC data for *C. albicans* are summarized in Table 1.

**Table 1.** Planktonic MIC (PMIC) characteristics for *Candida albicans* (*n* = 113)

Parameter	Rezafungin	Caspofungin	Amphotericin B	Fluconazole
PMIC range	<0.03 to 0.125	<0.03 to 0.5	<0.03 to 16	<0.125 to 8
PMIC_50_	0.03	0.03	0.25	<0.125
PMIC_90_	0.125	0.25	0.5	0.25

A similar level of activity was observed for *N. glabratus* (*n* = 34). Rezafungin again showed the lowest MICs (MIC_50_ ≤ 0.03 mg/L; MIC_90_ = 0.06 mg/L), followed by caspofungin and amphotericin B. Fluconazole was least active against this species, with MIC_50_ and MIC_90_ values of 4 and 8 mg/L, respectively. These data are presented in Table 2.

**Table 2.** Planktonic MIC (PMIC) characteristics for *Nakaseomyces glabratus* (*n* = 34)

Parameter	Rezafungin	Caspofungin	Amphotericin B	Fluconazole
PMIC range	<0.03 to 2	0.125 to 1	0.25 to 1	4 to >16
PMIC_50_	0.03	0.25	0.5	4
PMIC_90_	0.125	0.25	0.5	16

For *C. tropicalis* (*n* = 10), both rezafungin and caspofungin exhibited high activity, with MIC_50_ values of 0.03 mg/L and narrow MIC ranges. Amphotericin B showed moderate activity, whereas fluconazole did not demonstrate measurable inhibitory activity within the concentration range tested. Planktonic MICs for *C. tropicalis* are shown in Table 3.

**Table 3.** Planktonic MIC (PMIC) characteristics for *Candida tropicalis* (*n* = 10)

Parameter	Rezafungin	Caspofungin	Amphotericin B	Fluconazole
PMIC range	0.03–0.06	0.03–0.06	0.125–0.5	>16
PMIC_50_	0.03	0.03	0.25	>16
PMIC_90_	0.03	0.06	0.5	>16

In contrast, *C. parapsilosis* isolates (*n* = 10) were less susceptible to echinocandins. Both caspofungin and rezafungin showed elevated MICs compared with other species, whereas amphotericin B was the most active agent, with MIC_50_ and MIC_90_ both 0.125 mg/L. Fluconazole demonstrated the weakest activity against this species. These results are summarized in Table 4, with individual isolate MIC distributions shown in Figure S1 (available as Supplementary data at *JAC* Online) and individual MICs in Table S1.

**Table 4.** Planktonic MIC (PMIC) characteristics for *Candida parapsilosis* (*n* = 10)

Parameter	Rezafungin	Caspofungin	Amphotericin B	Fluconazole
PMIC range	0.25 to 2	0.25 to 1	0.125	0.5 to >16
PMIC_50_	1	1	0.125	2
PMIC_90_	2	1	0.125	4

### Rezafungin is effective at inhibiting *Candida* biofilm development

**Figure 1.**
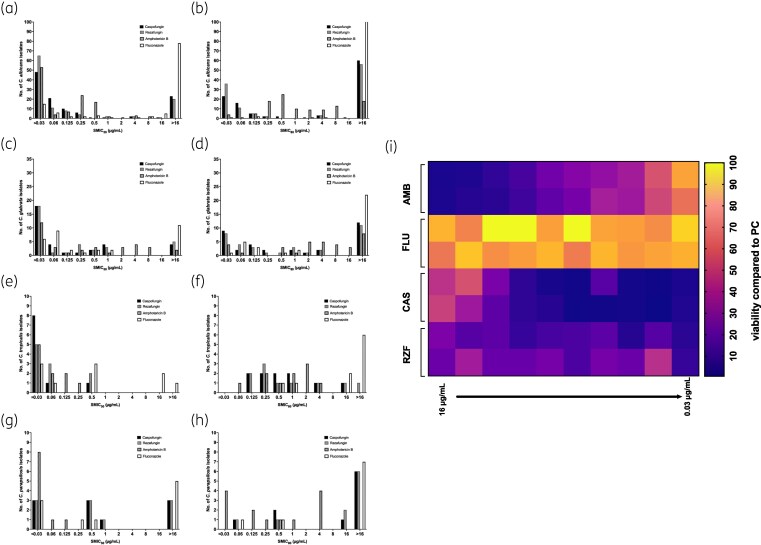
Sessile MICs (SMICs) of *Candida albicans* and *Candida glabrata* biofilms treated with CAS, RZF, AMB and FLU. *C. albicans* (a, b), *N. glabratus* (c, d), *C. tropicalis* (e, f) and *C. parapsilosis* (g, h) biofilms were treated with 0.03 to 16 mg/L of CAS, RZF, AMB and FLU. XTT metabolic reduction was used to calculate the SMIC_50_ (a, c, e, g) and SMIC_80_ (b, d, f, h) for each compound and for each isolate. Data are presented as histograms showing number of isolates at each concentration. The biofilm of the representative laboratory strain of *C. albicans*, SC5314, was formed for 24 h and treated with AMB, FLU, CAS and RZF for 24 h (0.03 to 16 mg/L). Anti-biofilm activity was quantified by a reduction in percentage XTT metabolism and presented in a heat map (i). Bright yellow indicates no reduction in viable cells and purple represents complete killing of viable cells. AMB, amphotericin B; CAS, caspofungin; FLU, fluconazole; RZF, rezafungin; XTT, 2,3-bis(2-methoxy-4-nitro-5-sulfophenyl)-2H-tetrazolium-5-carboxanilide salt.

Analysis of the SMIC_50_ profiles showed that the majority of isolates (58%) were susceptible to ≤0.03 mg/L rezafungin, whereas amphotericin B and caspofungin were equivalently effective for only 47% and 42% of these *C. albicans* isolates, respectively (Figure 1a). Fluconazole was generally ineffective for *C. albicans* biofilms, with 69% of isolates showing an SMIC_50_ >16 mg/L. With a more stringent assessment of antifungal activity, the SMIC_80_ was evaluated (80% metabolic inhibition). It was shown that rezafungin was effective at ≤0.03 mg/L, inhibiting metabolism for 32% of the isolates, followed by caspofungin (20%). Amphotericin B was effective across a range of concentrations, ranging from 0.25 to 16 mg/L, whereas and fluconazole were generally ineffective, with the majority of isolates (96%) exhibiting an SMIC_80_ >16 mg/L (Figure 1b).

For *N. glabratus* biofilm testing the majority of isolates (53%) were equivalently susceptible to ≤0.03 mg/L of rezafungin and caspofungin based on SMIC_50_ levels. Amphotericin B was also effective against 35% of the isolates at this concentration, whereas fluconazole was effective against 18% (Figure 1c). Generally, fluconazole was ineffective against these biofilms, with 32% of isolates unable to reduce metabolism by 50% at >16 mg/L. When the SMIC_80_ levels were assessed on these same isolates, caspofungin and rezafungin remained effective at ≤0.03 mg/L for 26% and 25% of the isolates, respectively. For amphotericin B only 12.5% of isolates were susceptible at this level. Fluconazole was ineffective, with 69% of isolates being unaffected by 16 mg/L. A large number of isolates were also unaffected by caspofungin (35%), rezafungin (32%) and amphotericin B (25%) at 16 mg/L. Amphotericin B was dispersed across a range of concentrations, ranging from 0.5 to 16 mg/L (Figure 1d).

Based on SMIC_50_ analysis, all *C. tropicalis* isolates (*n* = 10) were susceptible to caspofungin, rezafungin and amphotericin B, whereas fluconazole showed limited activity. Caspofungin reduced biofilm metabolic activity in 80% of isolates at ≤0.03 mg/L, compared with 50% for both rezafungin and amphotericin B and 30% for fluconazole (Figure 1e). Under SMIC_80_ conditions, no isolate was inhibited at 0.03 mg/L; caspofungin, rezafungin and amphotericin B achieved 80% metabolic reduction at 0.125–16 mg/L, whereas 60% of isolates exhibited SMIC_80_ values >16 mg/L for fluconazole (Figure 1f).

For *C. parapsilosis*, amphotericin B showed superior antibiofilm activity. At the SMIC_50_, 80% of isolates were inhibited at ≤0.03 mg/L, compared with 30% for caspofungin, rezafungin and fluconazole (Figure 1g). SMIC_50_ analysis confirmed reduced efficacy of echinocandins and fluconazole, with 60%, 60% and 70% of isolates, respectively, failing to reach 80% metabolic inhibition, whereas amphotericin B retained activity in 40% of isolates at ≤0.03 mg/L (Figure 1h).

Figure 1i acts an exemplar to illustrate *C. albicans* SC5314 treated with varying concentrations of amphotericin B, fluconazole, caspofungin and rezafungin. These data demonstrate that rezafungin and caspofungin are relatively more effective than amphotericin B and fluconazole.

### Rezafungin is non-inferior to caspofungin against high biofilm-forming strains

**Figure 2.**
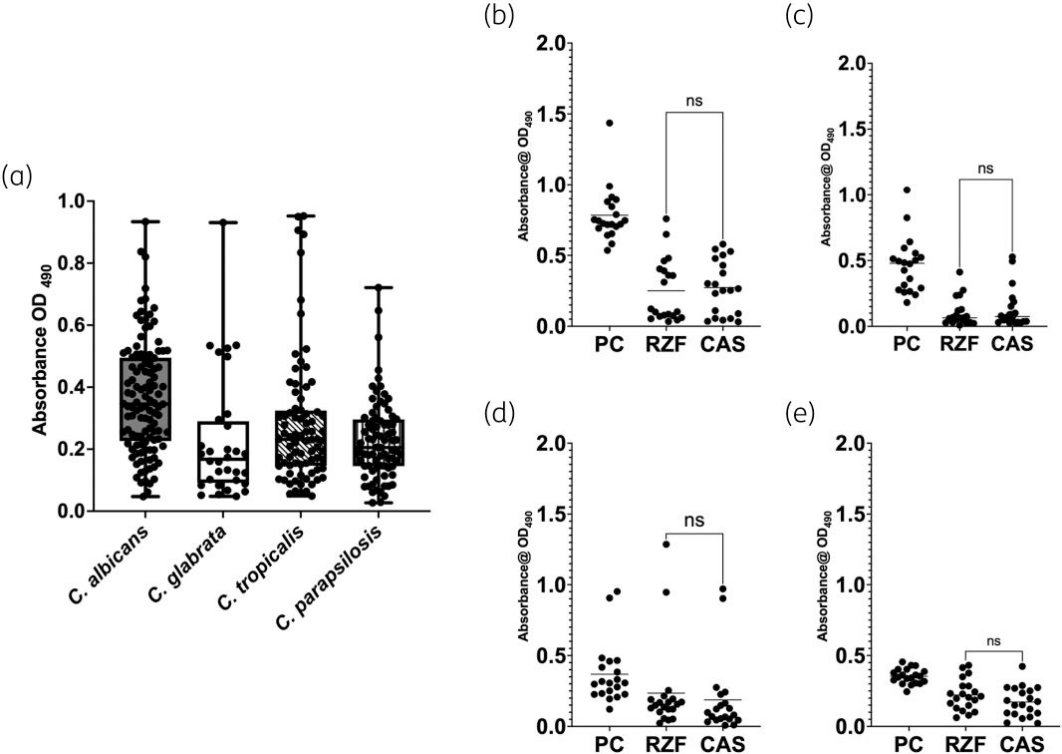
Assessment of anti-biofilm activity of CAS and RZF against low (LBF) and high (HBF) biofilm-forming clinical isolates. Biofilms of all *C. albicans*, *N. glabratus*, *C. tropicalis* and *C. parapsilosis* clinical strains were formed over 24 h and quantified by XTT metabolic reduction (a). Twenty HBF *C. albicans* (b), *N. glabratus* (c), *C. tropicalis* (d) and *C. parapsilosis* (e) clinical isolates were then selected and treated with CAS and RZF at 0.03 mg/L. Biofilm metabolism was quantified by XTT metabolic reduction for eight replicate biofilms. Independent experiments were performed in triplicate. CAS, caspofungin; PC, positive control; RZF, rezafungin; XTT, 2,3-bis(2-methoxy-4-nitro-5-sulfophenyl)-2H-tetrazolium-5-carboxanilide salt. A t-test was performed to compare CAS and RZF. Non-significance (ns) is denoted for each comparions (thin horizontal lines). PC denoted positive control.

Figure 2a illustrates the levels of biofilm heterogeneity observed spectroscopically. Overall, *C. albicans* formed significantly more robust biofilms (based on 3D architecture and hyphae) than *N. glabratus*, *C. parapsilosis* (*P* > 0.0001) and *C. tropicalis* (*P* = 0.0003). Absorbance values also exhibited a wider range for *C. albicans* (OD_490_ = 0.05 to 1.44) compared with *N. glabratus* (OD_490_ = 0.05 to 0.88), *C. parapsilosis* (OD_490_ = 0.027 to 0.721) and *C. tropicalis* (OD_490_ = 0.049 to 0.952).

The top 20 biofilm formers, determined to be the most robust based on XTT (Figure 2b) and visual inspection, were evaluated for their relative efficacy at 0.03 mg/L rezafungin and caspofungin. For *C. albicans* both rezafungin and caspofungin displayed significant anti-biofilm activity (*P* < 0.0001), though there was no significant difference between their activity at this concentration (*P* = 0.73; difference between means = 0.023; 95% CI, −0.11 to 0.16; Figure 2b). For *N. glabratus*, which forms physically less complex biofilms (monolayer clumps of yeasts), a similar trend was observed where rezafungin and caspofungin displayed significant anti-biofilm activity (*P* < 0.0001), but no significant difference was observed between rezafungin and caspofungin (*P* = 0.47; difference between means = 0.03; 95% CI,  −0.05 to 0.11; Figure 2c). No difference between the two antifungals was also observed for both *C. tropicalis* (*P* > 0.999; difference between means = −0.04; 95% CI,  −0.2 to 0.13; Figure 2d) and *C. parapsilosis* (*P* > 0.211; difference between means = −0.04; 95% CI, −0.11 to 0.01; Figure 2e).

**Figure 3.**
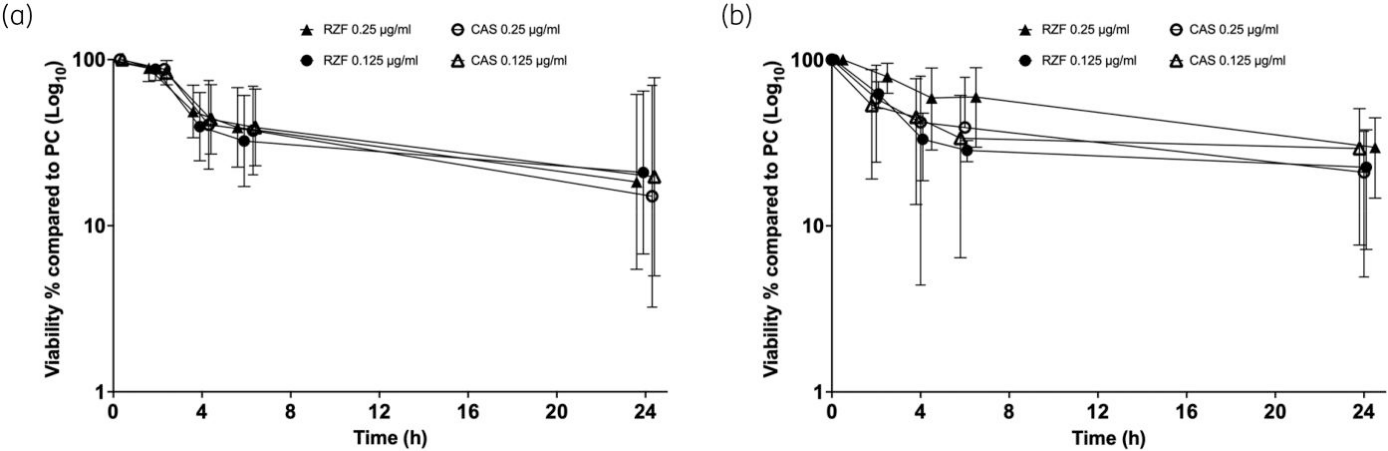
Time–kill studies (metabolic activity) of *Candida albicans* with CAS and RZF. Biofilms of five LBF (a) and HBF (b) strains were formed over 24 h and then treated with CAS or RZF at PMIC_90_ levels or 2 × PMIC_90_. Biofilms were washed and XTT metabolic reduction quantified after 2, 4, 6 and 24 h. Data were log_10_ transformed and a two-way ANOVA performed. Five biological replicates per isolate were assessed. CAS, caspofungin; HBF, high biofilm-forming; LBF, low biofilm-forming; PC, positive control; PMIC, planktonic MIC; RZF, rezafungin; XTT, 2,3-bis(2-methoxy-4-nitro-5-sulfophenyl)-2H-tetrazolium-5-carboxanilide salt. Error bars represent standard deviaton from mean.

We next evaluated how comparatively quickly rezafungin and caspofungin killed biofilms at 2, 4, 6 and 24 h using XTT on 10 strains (5 HBF and 5 LBF) at 0.125 and 0.25 mg/L (1× and 2× PMIC_90_). These data showed some variation between strain (Figure S2), though when combined analysis of log_10_-transformed data demonstrated and concentration dependent effects, but with no significant difference (*P* > 0.05) between treatments for both HBFs and LBFs (Figure 3). Comparison of the LBF and HBF strains revealed distinct response phenotypes. Although metabolic reduction was time dependent in both cases, the LBF phenotype killing was more rapid and uniform (Figure 3a), whereas the HBF was more delayed (Figure 3b), consistent with a more tolerant biofilm phenotype.

**Figure 4.**
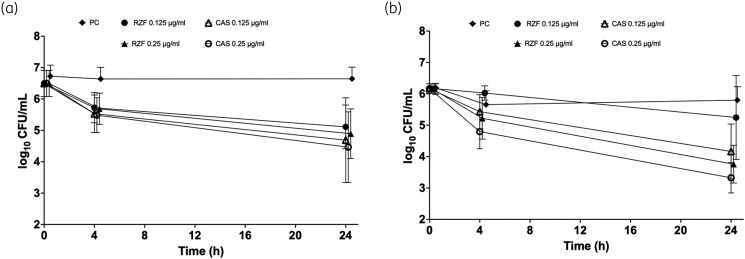
Time–kill studies (viable counts) of *Candida albicans* with CAS and RZF. Biofilms of two HBF and two LBF strains were formed over 24 h on Thermonox™ coverslips and treated with CAS or RZF at PMIC_90_ levels or 2 × PMIC_90_. Viable cells (cfu/mL) were quantified by Miles and Mirsa testing at 4 and 24 h. Data were log_10_ transformed and a two-way ANOVA performed. Three biological replicates per isolate were assessed. CAS, caspofungin; HBF, high biofilm-forming; LBF, low biofilm-forming; PC, positive control; PMIC, planktonic MIC; RZF, rezafungin. Error bars represent the standard deviation from the mean.

To validate these data, 10 *C. albicans* isolates were evaluated using total viable cell counting, where similar trends were observed, with a high level of consistency between XTT and cfu data (Figure 4). Again, time and concentration accounted for the greatest effects for both LBFs and HBFs. Although there was individual strain variation (Figure S3), there was no significant difference between the two antifungals, although caspofungin trended to be marginally more effective after 24 h of exposure. Comparison of the two datasets demonstrated notable differences in antifungal response profiles. Whereas killing was time dependent for both LBFs and HBFs, LBFs (yeast-phenotype) retained substantially higher viable burdens at 24 h compared with HBFs.

### Rezafungin displays superior biofilm inhibition characteristics to caspofungin

**Figure 5.**
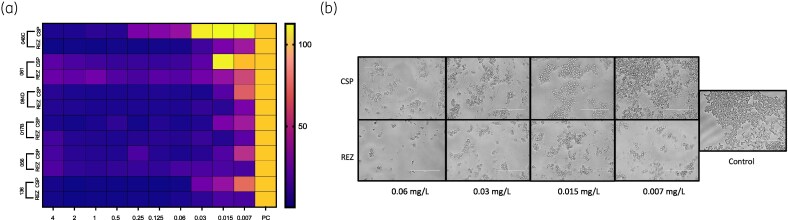
*Candida albicans* biofilm inhibition by RZF and CAS of low and high biofilm-forming isolates. (a) Heatmap representing three low biofilm isolates (048C, 061 and 064D) and three high biofilm isolates (017B, 056 and 136) exposed to serial doubling dilutions of RZF and CAS (0.007 to 4 mg/L). Bright yellow indicates no reduction in viable cells and purple represents complete killing of viable cells. (b) Microscopic evaluation of *C. albicans* strain 061 (low biofilm former) at ×40 magnification shows a comparative effect of RZF to CAS, where RZF induces reduced numbers of cellular biofilm aggregates on the surface. Scale bar = 100 μm. CAS, caspofungin; RZF, rezafungin.

Finally, we investigated the impact of rezafungin and caspofungin against the ability to inhibit biofilm formation using both high (SBS017B, SBS066, SBS136) and low (SBS048C, SBS061, SBS084) biofilm formers, evaluated using a biomass reading. A heat map was created (Figure 5a), and biofilm inhibition MICs are presented in Table 5. The heatmap shows the maximal biofilm growth in yellow, whereas biofilm inhibition is purple. Rezafungin was more effective at inhibiting biofilm development of *C. albicans* by greater than 80% for four of the six isolates tested. When this was visualized by microscopy it was evident that there was a concentration-dependent effect for rezafungin and caspofungin, but that biofilm formation was defined by smaller clusters of cells for rezafungin treatment compared with caspofungin (Figure 5b).

**Table 5.** Biofilm inhibition MICs

*C. albicans* strain	MBIC_80_ caspofungin, mg/L	MBIC_80_ rezafungin, mg/L
048C (LBF)	0.5	0.03
084D (LBF)	0.03	0.03
061 (LBF)	0.06	0.06
017B (HBF)	0.03	0.03
056 (HBF)	0.03	0.015
136 (HBF)	0.015	0.015

HBF, high biofilm former; LBF, low biofilm former; MBIC, minimum biofilm inhibitory concentration.

## Discussion

The data generated in this *in vitro* biofilm modelling study demonstrated that rezafungin exhibited non-inferior anti-biofilm activity compared with caspofungin against a large panel of *C. albicans*, *N. glabratus*, *C. tropicalis* and *C. parapsilosis* clinical isolates. We first demonstrated that rezafungin exhibits strong activity against planktonic cells of *C. albicans*, *C. glabrata* and *C. tropicalis*, with notably low MIC_50_ and MIC_90_ values, consistent with previously reported data on its broad-spectrum antifungal efficacy.^20,21,28^ In contrast, *C. parapsilosis* showed relatively high PMIC values (compared with other *Candida* spp.) for both caspofungin and rezafungin. These findings align with clinical data showing rezafungin to be non-inferior to caspofungin in treating candidaemia and invasive candidiasis.^18,19^ Notably, however, rezafungin maintained excellent antifungal activity against biofilm-associated cells.

With the exception of *C. parapsilosis*, our SMIC analysis revealed that a larger proportion of *C. albicans*, *C. glabrata* and *C. tropicalis* isolates were susceptible to low concentrations of rezafungin compared with caspofungin, with both superior to fluconazole and amphotericin B. This is consistent with the echinocandin class in general, which are highly active anti-biofilm agents that favour positive clinical outcomes compared with other antifungal classes.^3,4^ This underscores the importance of echinocandins in this context,^11,12,16^ but with the caveat that frequency of dosage is different between these two echinocandins, with rezafungin providing the same, if not better, anti-biofilm outcome compared with caspofungin. This provides an obvious advantage for the use of rezafungin.

A key feature of *Candida* spp. biofilms is their inherent heterogeneity.^8^  *C. albicans* and *N. glabratus* have quite distinct biofilm structures, with *C. albicans* exhibiting a vast 3D structure of intertwined hyphae and larger yeasts cells, whereas *N. glabratus* biofilms are defined by aggregated clusters of smaller yeasts cells. *C. tropicalis* and *C. parapsilosis* are intermediate versions of these;^2^ they are less complex, though generally more numerically dense with cells that lack elevated 3D structure. We and others have shown how biofilm forming phenotypes (low and high biofilm) differentially respond to antifungal therapy.^4,6,29^ Despite *C. albicans* having the ability to form hyphae, some strains are deficient in hyphal formation, and are instead like *N. glabratus*, and are generally considered LBFs *in vitro*. In this study, both species of *Candida*, irrespective of biofilm phenotype, showed similar response profiles to the anti-biofilm rezafungin and caspofungin, as shown in Figure 2. Of note, the observed paradoxical effect at higher echinocandin concentrations, a well-documented phenomenon in *Candida* spp., was also observed for both rezafungin and caspofungin. Whether this is due to limits of detection of the XTT metabolic assay (particularly for LBF strains) or a strain-dependent phenomenon remains to be elucidated. Although not explicitly the focus of this study, it underscores the need for optimal dosing strategies and further mechanistic studies. Aneuploidy may explain this phenomenon, as has been hypothesized,^30^ but data from this study and others indicate that lower concentrations of rezafungin and caspofungin are able to suppress and kill thicker and denser biofilms. We therefore took subsets of HBFs from *C. albicans* and *N. glabratus*, and were still able to show significant reductions of both species by rezafungin and caspofungin. Although no significant difference between these antifungals was observed, the trend indicated that rezafungin was more active. Investigating this further through time–kill studies showed strain-specific effects, but with no real discernible differences between each compound. It is noteworthy that viable cell reduction by cfu largely mirrored XTT, indicating that metabolic reduction is a valuable technique to assay antifungal efficacy for each strain.^31^

Finally, a key observation was that rezafungin exhibited superior ability to inhibit biofilm formation across both high and low biofilm-forming *C. albicans* isolates, albeit in a small subset (*n* = 3) of each phenotype. These results were supported by both quantitative XTT data and visualization via microscopy, which indicated a concentration-dependent effect and fewer, smaller biofilm clusters in rezafungin-treated wells. These findings align with earlier *in vitro* studies that demonstrated rezafungin's capacity to prevent biofilm establishment.^20^ Future clinical studies should aim to validate these *in vitro* findings and further assess rezafungin's efficacy in the context of persistent or device-associated candidaemia.

Our study is limited through the use of an *in vitro* biofilm model, which does not fully recapitulate the host environment or account for immune interactions. Also, although our strain panel was large and diverse, additional testing of non-*albicans Candida* species and clinically resistant isolates would further enhance our understanding of rezafungin's utility, especially at concentrations that simulate *C*_avg_, *C*_min_ and *C*_max_ concentrations. It is also critical that we further explore the mechanism of biofilm inhibition, in which future transcriptomic or proteomic analyses could elucidate the pathways modulated by rezafungin treatment.

### Conclusions

Our large-scale screening approach provides a robust validation across a diverse set of clinical isolates that rezafungin and caspofungin outperformed fluconazole and amphotericin B in terms of anti-biofilm activity. Moreover, analysis of biofilm inhibition showed that rezafungin was superior to caspofungin at preventing biofilm growth. Our findings support the hypothesis that rezafungin demonstrates potent anti-biofilm properties and performs at least as effectively as caspofungin in both biofilm inhibition and eradication assays. Together, these data indicate that rezafungin should be advocated for the clinical management of *Candida* spp. biofilm infections. Indeed, a recent meta-analysis of 18 clinical studies where biofilm-related infections were present (implant, endocarditis and osteoarticular infections) showed that rezafungin provided a successful outcome for 83.3% of patients over a 3 month duration.^32^ Given its differentiated pharmacokinetic/pharmacodynamic profile, once-weekly administration and promising *in vivo* activity, rezafungin represents a suitable candidate for broader use in the management of biofilm-associated *Candida* infections.

## Supplementary Material

dkag058_Supplementary_Data

## Supplementary data

Figures S1 to S3 and Table S1 are available as Supplementary data at *JAC* Online.
