# Supplementary material for: Rezafungin exhibits anti-biofilm properties against fungal biofilms in vitro
Source: J Antimicrob Chemother. 2026 Mar 5;81(4):dkag058. doi: 10.1093/jac/dkag058 (PMC13017008; doi:10.1093/jac/dkag058)

**Supplementary Table 1: Planktonic minimum inhibitory concentrations of four antifungals against Candidemia study blood isolates (**concentration range between 16 to 0.03 ug/mL**):**

| ***Candida* species** | **Amphotericin B (pMIC)** | **Fluconazole**  **(pMIC)** | **Caspofungin**  **(pMIC)** | **Rezafungin**  **(pMIC)** |
| --- | --- | --- | --- | --- |
| ***C. albicans*** |  |  |  |  |
| 003B | 0.125 | <0.125 | 0.125 | <0.03 |
| 011 | 0.5 | >16 | 0.125 | 0.06 |
| 013 | 0.125 | >16 | 0.125 | 0.06 |
| 014 | 0.125 | >16 | 0.125 | <0.03 |
| 015 | 0.5 | >16 | 0.06 | <0.03 |
| 016B | 0.125 | >16 | 0.06 | <0.03 |
| 016C | 0.125 | >16 | 0.06 | <0.03 |
| 017A | 0.125 | >16 | 0.06 | <0.03 |
| 017B | 0.5 | >16 | 0.06 | <0.03 |
| 020 | 0.125 | 0.25 | 0.125 | 0.125 |
| 003A | 0.125 | <0.125 | 0.25 | <0.03 |
| 003C | 0.25 | >16 | 0.06 | <0.03 |
| 003D | 0.25 | >16 | 0.125 | <0.03 |
| 004 | 0.06 | <0.125 | 0.25 | 0.06 |
| 002A | 0.03 | >16 | 0.25 | <0.03 |
| 002B | 0.25 | >16 | 0.125 | <0.03 |
| 016A | 0.125 | >16 | 0.06 | <0.03 |
| 009 | 0.125 | 0.25 | 0.5 | 0.125 |
| 024 | 0.25 | <0.125 | 0.03 | <0.03 |
| 025 | 0.06 | <0.125 | 0.25 | 0.125 |
| 026 | 0.25 | 0.5 | 0.5 | 0.25 |
| 030 | 0.25 | 0.25 | 0.5 | 0.06 |
| 031 | 0.03 | 0.25 | 0.06 | <0.03 |
| 032 | 0.06 | <0.125 | 0.25 | 0.125 |
| 033 | 0.125 | <0.125 | 0.5 | 0.125 |
| 034 | 0.25 | <0.125 | 0.25 | <0.03 |
| 035 | 0.25 | <0.125 | 0.25 | <0.03 |
| 036 | 0.25 | <0.125 | 0.5 | 0.125 |
| 037 | 0.25 | <0.125 | 0.25 | <0.03 |
| 038 | 0.25 | <0.125 | 0.25 | 0.06 |
| 039 | 0.25 | <0.125 | 0.06 | <0.03 |
| 44A | 0.25 | <0.125 | 0.06 | <0.03 |
| 44B | 0.25 | <0.125 | 0.06 | <0.03 |
| 44C | 0.25 | <0.125 | 0.03 | <0.03 |
| 45A | 0.5 | <0.125 | 0.03 | <0.03 |
| 45B | 0.25 | <0.125 | 0.03 | <0.03 |
| 48C | 0.5 | 0.25 | 0.25 | 0.125 |
| 48E | 0.25 | 0.25 | 0.125 | 0.06 |
| 048G | 0.25 | 0.25 | 0.25 | 0.125 |
| 048J | 0.5 | 0.25 | 0.5 | 0.25 |
| 056 | 0.5 | <0.125 | 0.06 | <0.03 |
| 059 | 0.5 | <0.125 | 0.03 | <0.03 |
| 060 | 0.25 | <0.125 | 0.06 | <0.03 |
| 061 | 0.25 | <0.125 | 0.125 | 0.125 |
| 063A | 0.25 | <0.125 | 0.06 | <0.03 |
| 069A | 0.5 | 0.25 | 0.06 | 0.06 |
| 070 | 0.25 | <0.125 | 0.03 | <0.03 |
| 071 | 0.5 | <0.125 | 0.06 | 0.06 |
| 072 | 0.25 | 0.25 | 0.03 | <0.03 |
| 073 | 0.5 | <0.125 | 0.06 | <0.03 |
| 074 | 0.5 | <0.125 | 0.03 | <0.03 |
| 075 | 0.5 | <0.125 | 0.03 | <0.03 |
| 081 | 0.5 | <0.125 | 0.06 | 0.06 |
| 084A | 0.5 | <0.125 | 0.03 | <0.03 |
| 084B | 0.25 | <0.125 | 0.06 | <0.03 |
| 084C | 0.25 | <0.125 | 0.03 | <0.03 |
| 084D | 0.25 | <0.125 | 0.03 | <0.03 |
| 084E | 0.25 | <0.125 | 0.03 | <0.03 |
| 087A | 0.5 | <0.125 | 0.03 | <0.03 |
| 087B | 0.5 | <0.125 | 0.03 | <0.03 |
| 088A | 4 | <0.125 | 0.03 | <0.03 |
| 088B | 4 | <0.125 | 0.03 | <0.03 |
| 088C | 4 | <0.125 | 0.03 | <0.03 |
| 089A | 2 | <0.125 | 0.03 | <0.03 |
| 089B | 2 | <0.125 | 0.03 | <0.03 |
| 092 | 8 | <0.125 | 0.03 | <0.03 |
| 093G | 4 | <0.125 | 0.06 | <0.03 |
| 094 | 8 | <0.125 | 0.03 | <0.03 |
| 099A | 4 | <0.125 | 0.03 | <0.03 |
| 099B | 8 | <0.125 | 0.03 | <0.03 |
| 100A | 4 | <0.125 | 0.03 | <0.03 |
| 100B | 4 | <0.125 | 0.03 | <0.03 |
| 100C | 4 | <0.125 | 0.03 | <0.03 |
| 103 | 16 | <0.125 | 0.03 | <0.03 |
| 105 | 8 | <0.125 | 0.03 | <0.03 |
| 109 | 4 | <0.125 | 0.03 | <0.03 |
| 111 | 8 | <0.125 | 0.06 | <0.03 |
| 113 | 0.5 | <0.125 | 0.03 | <0.03 |
| 116 | 0.25 | 1 | 0.5 | <0.03 |
| 125 | 8 | <0.125 | 0.03 | <0.03 |
| 128 | 8 | <0.125 | 0.03 | <0.03 |
| 129 | 8 | 0.25 | 0.03 | <0.03 |
| 131 | 8 | <0.125 | 0.03 | <0.03 |
| 132 | 8 | <0.125 | 0.06 | <0.03 |
| 136 | 0.25 | 0.25 | 0.06 | <0.03 |
| 138 | 8 | 0.25 | 0.03 | <0.03 |
| 140 | 2 | <0.125 | 0.03 | <0.03 |
| 142 | 8 | 0.25 | 0.03 | <0.03 |
| 143 | 4 | <0.125 | 0.03 | <0.03 |
| 145 | 8 | 0.25 | 0.06 | <0.03 |
| 147 | 0.5 | <0.125 | 0.03 | <0.03 |
| 148 | 0.5 | <0.125 | 0.06 | <0.03 |
| 154 | 0.5 | <0.125 | 0.03 | <0.03 |
| 156 | 0.5 | <0.125 | 0.03 | <0.03 |
| 165 | 0.5 | <0.125 | 0.03 | <0.03 |
| 168 | 8 | <0.125 | 0.03 | <0.03 |
| 171 | 0.5 | <0.125 | 0.125 | <0.03 |
| 172 | 0.5 | <0.125 | 0.125 | <0.03 |
| 177A | 0.25 | <0.125 | 0.06 | <0.03 |
| 175 | 1 | <0.125 | 0.03 | <0.03 |
| 182 | 0.5 | <0.125 | 0.06 | <0.03 |
| 184 | 0.5 | 4 | 0.25 | <0.03 |
| 187 | 0.25 | <0.125 | 0.06 | <0.03 |
| 193A | 0.25 | <0.125 | 0.125 | <0.03 |
| 193B | 0.25 | <0.125 | 0.06 | <0.03 |
| 196 | 0.25 | 0.5 | 0.25 | 0.06 |
| 198 | 0.5 | <0.125 | 0.06 | <0.03 |
| 199 | 0.5 | 8 | 1 | 0.06 |
| 204 | 0.5 | 4 | 0.125 | <0.03 |
| 205 | 0.5 | 0.25 | 0.03 | <0.03 |
| 210 | >16 | <0.125 | >16 | >16 |
| 212A | 0.5 | <0.03 | 0.06 | <0.03 |
| 212B | 0.5 | <0.03 | 0.06 | <0.03 |
| **Non-*albicans*** |  |  |  |  |
| *C. glabrata* |  |  |  |  |
| 001 | 0.5 | 2 | 0.5 | <0.03 |
| 006 | 0.5 | 4 | 0.5 | <0.03 |
| 007 | 0.5 | 4 | 1 | <0.03 |
| 010 | 0.5 | 8 | 1 | <0.03 |
| 012 | 0.5 | 4 | 0.25 | <0.03 |
| 018A | 0.5 | 2 | 0.25 | <0.03 |
| 019 | 0.5 | 2 | 0.125 | <0.03 |
| 023 | 0.5 | 4 | 0.25 | <0.03 |
| 041 | 0.5 | 0.5 | 0.25 | 2 |
| 042 | 0.5 | 8 | 0.125 | <0.03 |
| 043 | 0.5 | 1 | 0.125 | <0.03 |
| 046 | 0.5 | 8 | 0.5 | <0.03 |
| 048A | 0.5 | 4 | 0.5 | <0.03 |
| 052 | 0.25 | 4 | 0.5 | <0.03 |
| 053 | 0.5 | 4 | 0.25 | <0.03 |
| 054 | 0.5 | 2 | 0.5 | <0.03 |
| 055 | 0.5 | 2 | 0.25 | <0.03 |
| 057 | 0.5 | 4 | 0.125 | <0.03 |
| 058 | 0.5 | 4 | 0.5 | 0.06 |
| 065 | 0.5 | 4 | 0.125 | 0.06 |
| 066 | 0.5 | >64 | 0.25 | <0.03 |
| 067 | 1 | 4 | 0.125 | 0.06 |
| 068A | 0.5 | 2 | 0.25 | 0.125 |
| 69B | 0.25 | 8 | 0.25 | 0.125 |
| 076 | 1 | 8 | 0.125 | <0.03 |
| 077 | 0.5 | 8 | 0.25 | 0.125 |
| 078 | 0.25 | 4 | 0.5 | <0.03 |
| 079 | 0.5 | 8 | 0.125 | <0.03 |
| 080 | 0.5 | 2 | 0.5 | <0.03 |
| 082 | 0.5 | 4 | 0.125 | <0.03 |
| 084F | 0.5 | 16 | 0.25 | <0.03 |
| 093A | 0.5 | 2 | 0.25 | 0.125 |
| 097A | 0.5 | 1 | 0.5 | <0.03 |
| 098 | 0.5 | 2 | 0.125 | 0.125 |
| *C. parapsilosis* |  |  |  |  |
| 152 | 0.125 | 2 | 1 | 2 |
| 158 | 0.125 | 2 | 1 | 1 |
| 040 | 0.125 | >16 | 0.25 | 0.25 |
| 062 | 0.125 | 1 | 1 | 1 |
| 029 | 0.125 | 4 | 1 | 2 |
| 126 | 0.125 | 4 | 1 | 1 |
| 027 | 0.125 | 2 | 1 | 1 |
| 091B | 0.125 | 2 | 1 | 2 |
| 155 | 0.125 | 0.5 | 1 | 1 |
| 135 | 0.125 | 1 | 1 | 2 |
| *C. tropicalis* |  |  |  |  |
| 179 | 0.5 | >16 | 0.03 | 0.03 |
| 095 | 0.5 | >16 | 0.03 | 0.03 |
| 051A | 0.25 | >16 | 0.03 | 0.03 |
| 188 | 0.5 | >16 | 0.03 | 0.03 |
| 149 | 0.25 | >16 | 0.06 | 0.06 |
| 160 | 0.25 | >16 | 0.06 | 0.03 |
| 141 | 0.5 | >16 | 0.03 | 0.03 |
| 209 | 0.25 | >16 | 0.06 | 0.03 |
| 161 | 0.25 | >16 | 0.03 | 0.03 |
| 028 | 0.125 | >16 | 0.03 | 0.03 |

**Supplementary Figure1: Planktonic minimum inhibitory concentrations of four antifungals against Candidemia study blood isolates (**concentration range between 16 to 0.03 ug/mL**):**


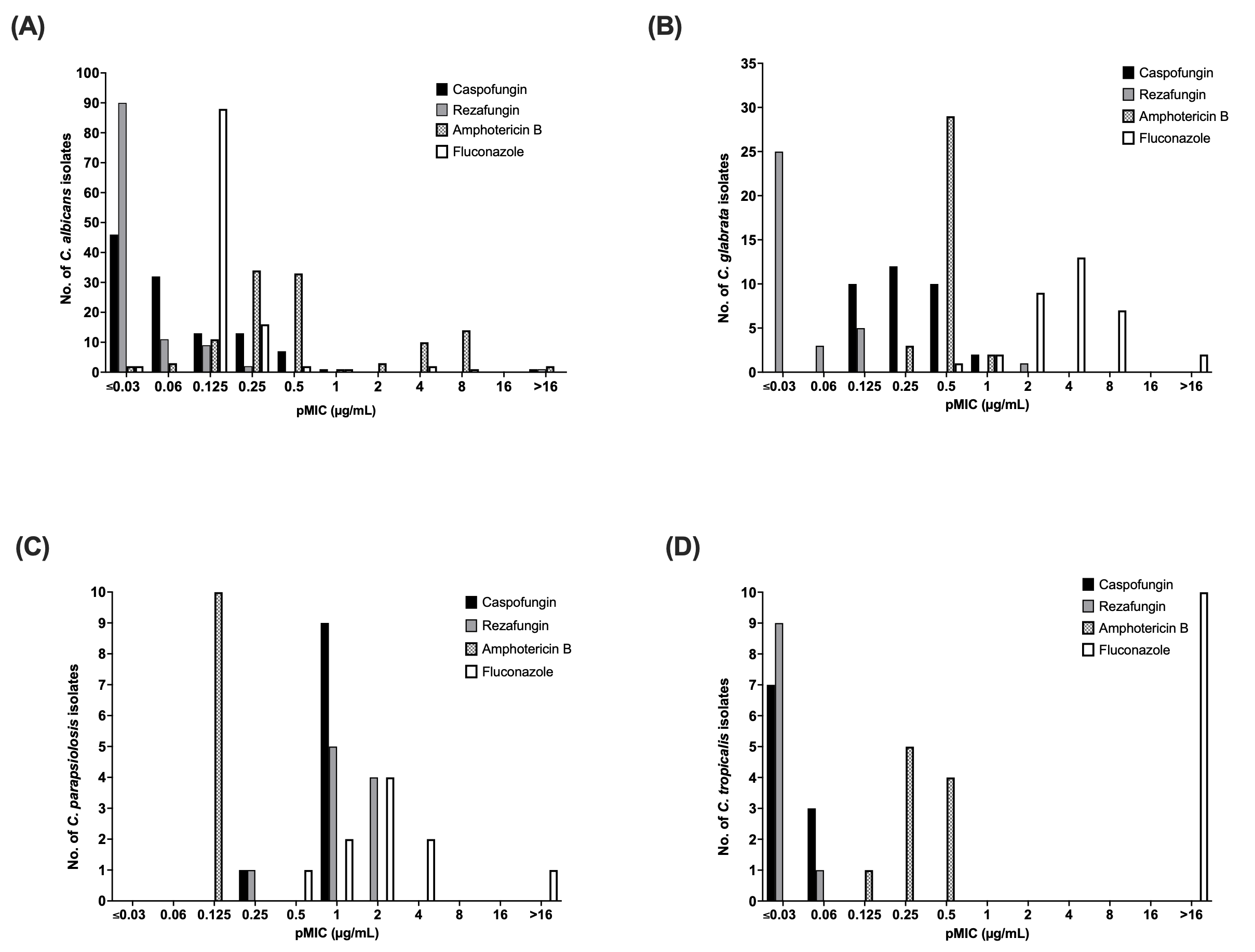


**Supplementary Figure 2: Time kill studies (XTT) of *Candida albicans* with CAS and RZF.** Biofilms of five LBF strains (A – 48C, B – 48E, C – 61, D – 84 , E - 184) and five HBF strains (F – 17B, G – 45B, H – 56, I – 136, J – 182) were formed over 24h on 96 well microtitre and treated with CAS or RZF at 0.125 ug/ml or 0.25 ug/ml. Metabolism was assessed by XTT at 0, 2, 4, 6 and 24h. Data was log_10_ transformed and a two-way ANOVA performed. Five biological replicates per isolate was assessed.


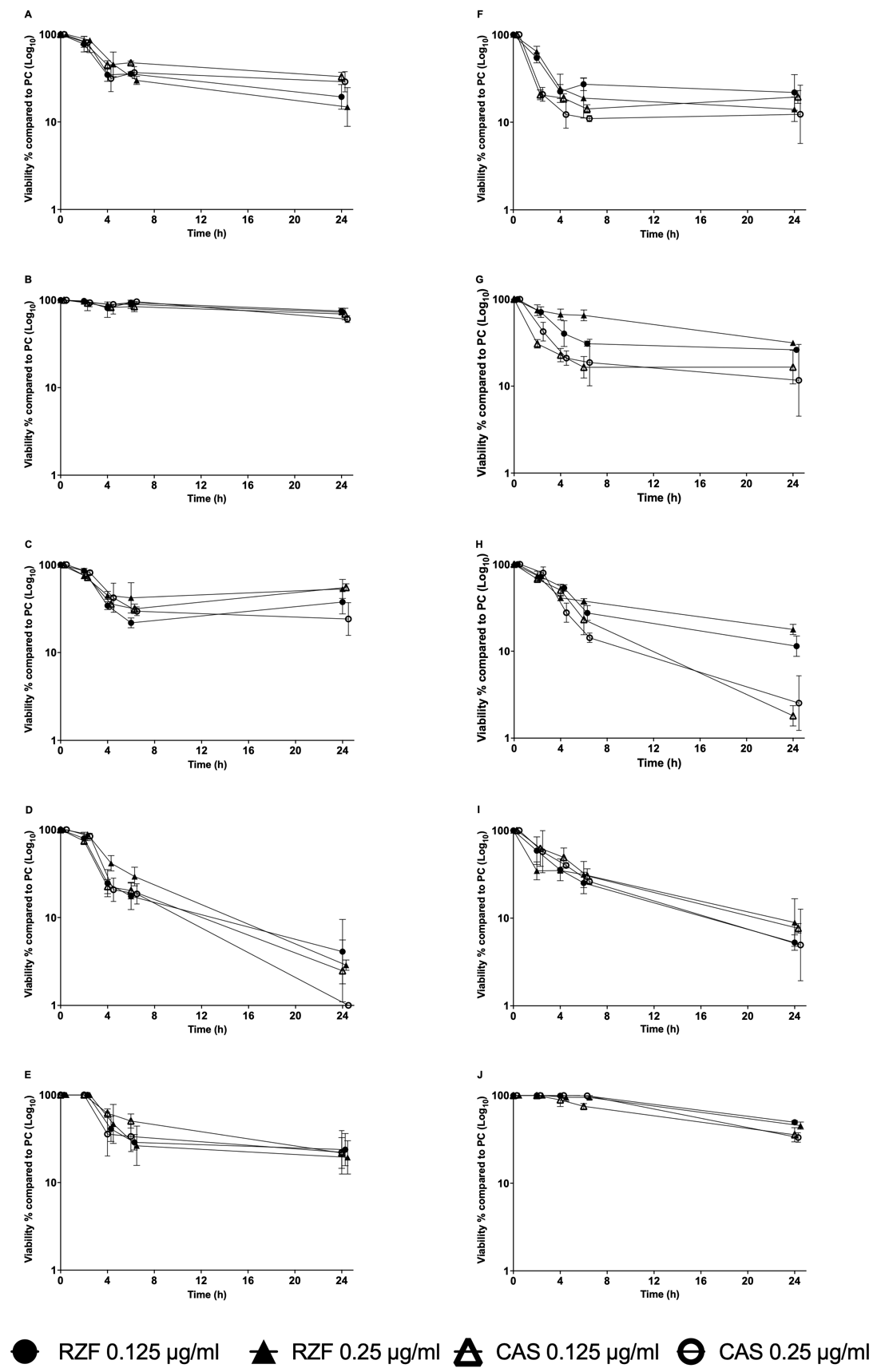


**Supplementary Figure 3: Time kill studies (cfu) of *Candida albicans* with CAS and RZF.** Biofilms of five LBF strains (A – 48C, B – 48E, C – 61, D – 84 , E - 184) and five HBF strains (F – 17B, G – 45B, H – 56, I – 136, J – 182) were formed over 24h on thermanox coverslips and treated with CAS or RZF at 0.125 ug/ml and 0.25 ug/ml. Viable cells (cfu/ml) were quantified by Miles and Mirsa testing at 4, and 24h. Data was log_10_ transformed and a two-way ANOVA performed. Three biological replicates per isolate was assessed.


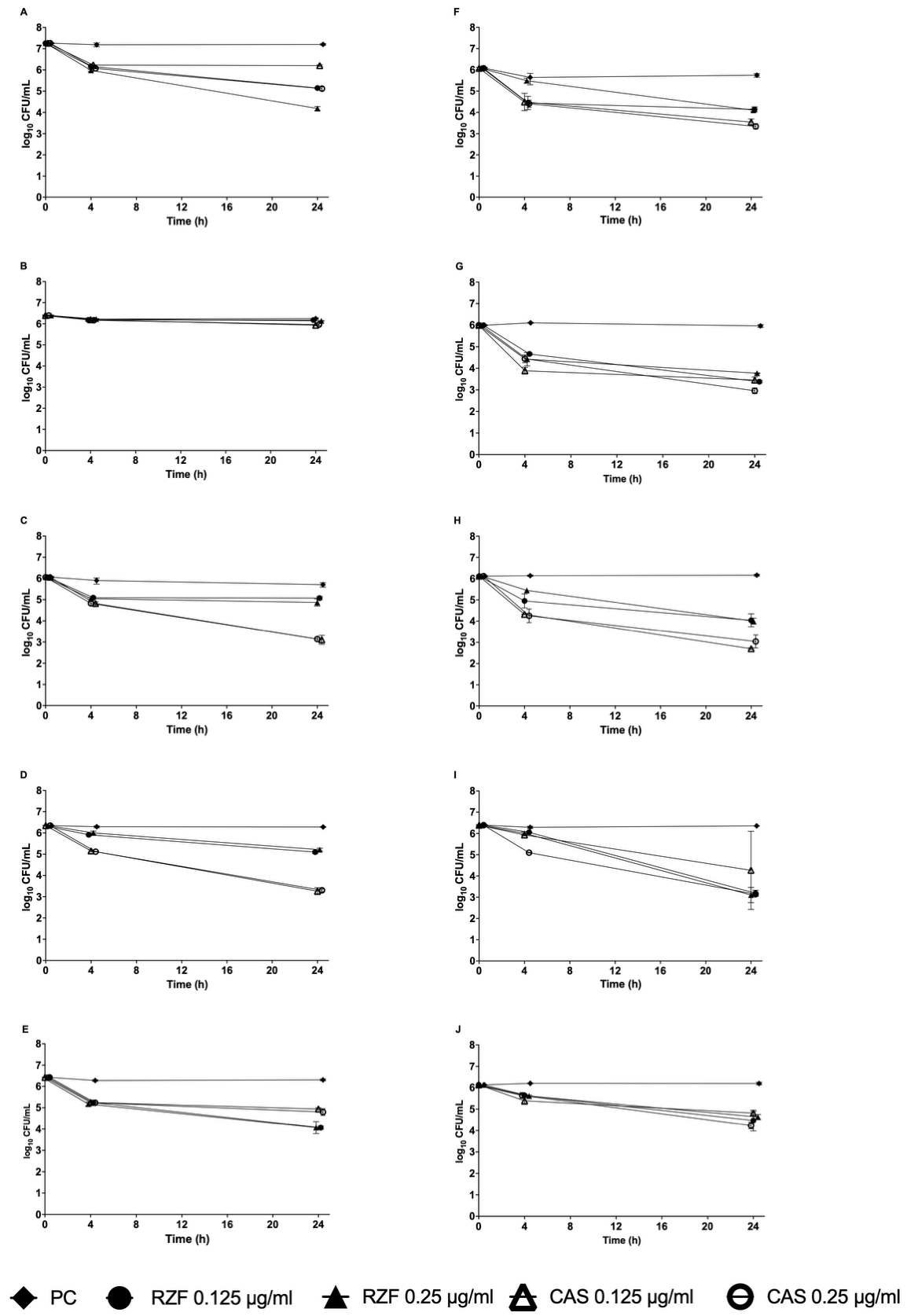

Supplement: dkag058_Supplementary_Data [file dkag058_supplementary_data.docx]
